# Evaluation of medical students of teacher-based and student-based teaching methods in Infectious diseases course

**Published:** 2015

**Authors:** I Ghasemzadeh, T Aghamolaei, F Hosseini-Parandar

**Affiliations:** * Infectious & Tropical Diseases Research Center, Hormozgan University of Medical Sciences, Bandar Abbas, Iran,; ** Department of Public Health, Health School, Hormozgan University of Medical Sciences, Bandar Abbas, Iran,; *** Department of Social Sciences, Shahid Bahonar University of Kerman, Kerman, Iran

**Keywords:** Problem-Based Learning, lectures, students, medical, communicable diseases

## Abstract

**Introduction:** In recent years, medical education has changed dramatically and many medical schools in the world have been trying for expand modern training methods. Purpose of the research is to appraise the medical students of teacher-based and student-based teaching methods in Infectious diseases course, in the Medical School of Hormozgan Medical Sciences University.

**Methods:** In this interventional study, a total of 52 medical scholars that used Section in this Infectious diseases course were included. About 50% of this course was presented by a teacher-based teaching method (lecture) and 50% by a student-based teaching method (problem-based learning). The satisfaction of students regarding these methods was assessed by a questionnaire and a test was used to measure their learning. information are examined with using SPSS 19 and paired t-test.

**Results:** The satisfaction of students of student-based teaching method (problem-based learning) was more positive than their satisfaction of teacher-based teaching method (lecture).The mean score of students in teacher-based teaching method was 12.03 (SD=4.08) and in the student-based teaching method it was 15.50 (SD=4.26) and where is a considerable variation among them (p<0.001).

**Conclusion:** The use of the student-based teaching method (problem-based learning) in comparison with the teacher-based teaching method (lecture) to present the Infectious diseases course led to the student satisfaction and provided additional learning opportunities.

## Introduction

Lecturing has been the main type of education for a long time. During recent decades, newer technologies have been implemented and visual aids such as slides and PowerPoint presentations have been used to boost education. Since lectures have a low effect on the development, the employment of newer techniques is inevitable and actively engage the students in the education process [**1**].

The main point that leads to an effective learning is an effective and high quality teaching in a proper environment. Making an efficient learning environment is one of the main challenges of medical education. The daily increase in the medical sciences leads to realization more problems. But then, this lecture based education has been replaced with student based education. This has provided new responsibilities for policy makers, professors and students According to selecting the most proper teaching and learning methods based on study field [**2**].

In an overall classification of teaching methods, those may divided into lecture based and student based methods. Group discussion & problem-solving are considered student based methods and lecturing is considered a lecture based method. In student based education, students face with an exciting challenge and brainstorm over solving the challenge. Irrelevant thoughts and ideas are filtered and the conversation goes on over the solutions. In this method, students face with the responsibility of the teaching process and attempt to realize the issue under the guidance of the professor [**3**]. 

One of the primary applications of the problem solving education is that as they recognize the educational purposes they learn both the basic and the clinical sciences [**2**,**4**]. This method improves motivation and clinical reasoning skills [**5**]. The students spend more time for self-teaching and implement various information origins such as libraries and digital libraries more frequently [**4**]. methodical study has shown, so the problem-solving education increases technical, social, cognitive, control, analysis, and cultural skills of the students [**6**]. 

Although newer teaching methods have been introduced, the lecture based method is still one of this countless usual methods since that was the safest and easiest method and the professor can become a real control over the class. Evidence showed that a decent lecture content by a noble lecturer may Leading to positive, appropriate and reasonable results. However, each method has its benefits and cons. Thus, everything those learning process elements (such as curriculum, teaching methods and students’ learning) should be evaluated [**7**]. This has led to various discussions and studies. For example, Carey conducted a descriptive examination to discover that students’ experiences during the problem solving method and its impacts on their gained knowledge. They showed that larger than 50 percent of those members described the process as “relatively hard”, however, improvement in knowledge was reported [**8**]. This research is led to appraise and compare that efficiency lecture-based method versus the student-based method among the medical students of Hormozgan University of Medical Sciences.

## Method

This interventional study was conducted on fourth year medical students of Hormozgan Medical Sciences University in 2013 during their infectious diseases course (three credits equaled to 51 educational hours). All the 52 scholars that got this course were enrolled.

Both the lecture based and the student-based methods were selected via the teaching style (50 percent each). In the lecture-based method, the professor presented a lecture by the support of PowerPoint slides. In the student-based method, the professor asked a problem or question in that start and students had to prepare themselves and study on the issue for the next session. During the following session, students mentioned on this issue and joined in a supervised discussion. Finally, the professor completed and concluded the discussions. 

Both methods were assessed with applying a Likert-based questionnaire. This application is produced with experts while using valid scientific sources. It included 19 questions. Each question contained five options including excellent (five points), good (four points), moderate (three points), weak (two points) and very weak (one point). Thus, each subject should be a score range of one to five. The questionnaire was trustworthy (Cronbach’s alpha = 0.89) & this validity was confirmed by experts.

The question sheet is given at the ending term and students are required for demonstrate their opinions. The students’ names were concealed. Also, in last exam, each student had two individual scores (out of 20) and the final scores of any way were compared. 

Data are recorded into spss v.19 and paired t-test was utilized to analyze the varieties of any process. 

## Results

All participants fully fill in and turned the questionnaires (response rate = 100%). Among all students, 46.2 percent are men and 53.8 percent were female.

According to the outcomes from the research, the variation among the lecture based method and the student based method was significant in 12 out of 19 questions (p<0.05). In eight of these items including the student’s participation in education, stability of education topics, self-confidence, personal difference consideration, long-term memory involvement, motivating students, interaction between professor and students, and creation of a team-work sense, the student based method is examined as the better method. On the other hand, the lecture-based method was better in four items; quality of education content, compliance to the structure and study sequence, efficacy of class time and creating a comfortable environment in the classroom. Also, this variation among seven items were not statistically significant; forming a positive attitude regarding study topics, establishing the professional requirements from that students, helping them to achieve the educational goals, a better perception of the subject contents, deeper understanding of the study contents, better responses to the questions and application of the education (**Table 1**).

The final score of the lecture based mode and that student-based method was 12.03 ± 4.08 and 15.50 ± 4.26, respectively. Paired t-test showed that this difference was significant (p<0.001, t= -8.82).

**Table 1:** Students’ attitude toward the two educational methods (paired t-test)

	average	scale variation	average	scale variation	T	P-value
Quality of study content	3.80	1.04	3.21	1.16	2.66	0.01*
Creating a positive attitude toward the study topics	3.48	0.99	3.48	1.09	0.000	1.00
Preparing professional needs of the students	3.57	1.09	3.32	1.21	1.01	0.31
Student participation during this training method	2.73	1.06	4.02	0.99	-5.63	0.000*
Helping scholars realize the instructional purposes	3.51	1.09	3.31	1.19	0.92	0.35
Better understanding of the study content	3.51	0.85	3.50	1.11	0.09	0.92
Long-term memory improvement	3.08	0.98	3.63	1.12	-2.51	0.01*
Motivating students to learn	2.96	0.96	3.46	1.19	-2.46	0.01*
Compliance to study structure and sequence	3.92	1.02	3.11	0.94	4.03	0.000*
Deeper understanding of the study content	3.26	0.95	3.46	1.07	-0.92	0.35
Better response to questions	3.40	0.91	3.46	1.05	-0.26	0.79
Stability of learning process	3.09	0.91	3.78	1.12	-3.06	0.003*
Application of the study content	3.40	1.15	3.55	1.10	-0.70	0.48
Self-confidence improvement	2.61	0.95	4.00	1.22	-6.06	0.000*
Considering the student differences	2.84	1.28	3.48	1.24	-2.65	0.01*
Better communication with scholars and teachers	2.94	0.97	3.78	1.28	-3.69	0.001*
Creating a sense of teamwork among students	2.42	0.95	3.80	1.10	-6.72	0.000*
Time efficacy	3.94	1.09	2.80	1.08	4.83	0.000*
Creating a comfortable environment	3.65	1.11	3.05	1.25	2.55	0.01*
* Significant at a 0.05 step						

## Discussion

The purpose of the research is to demonstrate the students’ attitude toward the lecture-based education and the student based education in the infectious diseases course of Hormozgan Medical Sciences University. Implementing newer educational techniques and improving them is a main goal in organizations that are involved in education. Most universities of the world are planning for improve theirs educational methods for develope this students’ learning. There was 2 main patterns; lecture based education and student based education. Problem solving is a type of student based education that leads to gaining professional views, communicational, proper Problem-solving and gaining knowledge skills [**9**].

The outcomes of the research revealed so the student based education leads to an improvement in student participance, better long-term memory, motivating students, stability of knowledge, higher self-confidence level, recognizing the educational differences, better student master interactions, and creating a sense of team-work. Also, learners got greater in this problem solving method topics in the final exam. These results were compatible with these findings of Nikfar et al. [**10**], Kermaniyan et al. [**11**], Momeni Danaei et al. [**12**], Jafari et al. [**13**], Hekmatpour et al. [**14**]. Another study also showed that students were more prone to group discussion and problem solving than to lecture [**15**]. Another study that enrolled the nursing the scholar explained that they favored group discussions [**16**]. Our results also showed that the student-based education was related to student satisfaction. This might be due to the students activating mind during the teaching, deeper knowledge, and assigning more time for studying. Also, these methods give more opportunities to the learner to review and criticize the lecturer and the educational contents [**17**-**19**].

The participants of this study believed that implementing the student based methods such as problem solving, results in higher contribution of students in the learning process and creates a sense of team group among students. This was consistent with Nikfar et al. [**10**] and Kermaniyan et al. [**11**]. Creating a sense of contribution is an important issue in improving the medical education and since the team work is a necessary part of problem solving, it can be expected that this method will reduce the personal differences and increase the student contribution in the team work [**20**].

Nikfar et al. [**10**] and Qin et al. [**21**] showed in their study that the student based educational methods increase the students’ interest and motivation. In fact, the problem solving method results in critical thinking and communicational skill development and increases the student’s interest in learning.

Participants demonstrated that the lecture based method outcome in a better study content quality, study sequence and structure and a higher time efficacy. Other studies have also mentioned that the scientific information are often unorganized [**11**,**22**]. In the lecture-based method, the professor has more authority in the classroom and can teach study content in a more organized and less time-consuming way. In this study, participants showed that a more comfortable environment was present in the lecture-based method. This was different from our expectations and further research is needed in that concern. 

The mean final exam score of the student-based method was importantly larger than that speech based method. Mahram et al. also conducted a study for contrast that lecture based method and group discussion method and showed no difference in their final scores [**23**]. Their results were inconsistent with our study. Delaram also compared these two methods among midwifery scholars confirmed no variation in final scores [**24**]. Momeni Danaei et al. [**12**] also revealed no important variation among the 2 learning styles. Dusold and Sadoski also led a learning via compare the final exams of students of both methods. They described no statistically important variation [**25**]. Herzig et al. also conducted that research on medical students during their pharmacology course. They also reported no important variation in none of the taken exams [**26**]. Safari et al. [**18**] explained that mean scores of the student based method is importantly larger than the lecture based method. Eslavin et al. also reasoned that this student based methods and combined models have a better impact on the learning of students’ [**27**]. The results of this 2 researches were consistent with our study. It must be held in memory that in our study, the study content of this 2 technique was different and this might be the reason for different scores. However, since the research findings are not consistent with most similar studies, more study is required. 

## Conclusion

It can be terminated that the overall satisfaction of students attending the infectious diseases course was higher and more learning opportunities were provided.

**Acknowledgement**

We would want to gratitude all the students who participated in the research.
